# A time-efficient protocol for transthoracic echocardiography during transfemoral transcatheter aortic valve implantation: early identification and effective management of intraprocedural complications

**DOI:** 10.1186/s44156-022-00005-6

**Published:** 2022-08-17

**Authors:** Panagiotis Savvoulidis, William E. Moody, Rick Steeds, Peter F. Ludman, Joseph R. Bradley, Aldrin Singh, Ewa Lawton, M. Adnan Nadir, Sagar N. Doshi

**Affiliations:** 1 Department of Cardiology, Queen Elizabeth Hospital Birmingham, Mindelsohn Way, Edgbaston, Birmingham, B15 2WB UK; 2 Institute for Cardiovascular Sciences, College of Medical & Dental Sciences, University of Birmingham, Edgbaston, Birmingham, B15 2TT UK

**Keywords:** Transcatheter aortic valve implantation, Echocardiography guidance, Complications

## Abstract

Transfemoral transcatheter aortic valve implantation (TAVI) under conscious sedation is the most widely used method of implantation. Echocardiography is used to detect complications and to assess the implantation result. The aim of this paper is to provide a time-efficient protocol when transthoracic echocardiography (TTE) is used to guide TAVI procedures.

**Supplementary Information:**

The online version contains supplementary material available at 10.1186/s44156-022-00005-6.

## Introduction

Transfemoral transcatheter aortic valve implantation (TAVI) undertaken with conscious sedation, rather than with general anaesthesia, is the most widely practised mode of implantation in Europe. Transthoracic echocardiography (TTE) is the main form of imaging employed in these procedures and is used to detect complications, assess the result of transcatheter heart valve (THV) implantation and guide further management. We propose a time-efficient, focused protocol for echocardiographers to follow when echocardiography is used to guide transfemoral TAVI undertaken with conscious sedation. This protocol provides guidance on which key assessments are required, important pitfalls and provides examples of important complications that may arise in order to allow timely identification and effective management. In the modern era of minimalist TAVI with transfemoral access and conscious sedation, transoesophageal echocardiography (TOE) may be preferable in a small proportion of TAVI procedures undertaken with general anaesthesia, with the benefit of continuous echocardiographic monitoring and reduced risk of contamination of the sterile field. TOE guidance may also be preferable in patients with severe renal insufficiency, particularly in conjunction with fusion imaging, to minimize contrast use and the consequent risk of renal injury.

## Background

Aortic stenosis is the most prevalent valvular lesion in Europe and North America[1, 2]. The first Transcatheter Aortic Valve Implantation (TAVI) procedure was performed in man in 2002[3] and transcatheter heart valves (THV) became commercially available in Europe in 2007 and in North America in 2011. TAVI has become the treatment of choice in patients considered inoperable and at high operative risk and is increasingly used in patients at intermediate and low risk, supported by guidelines[4, 5]. In the early years of TAVI, due to the large profile of the delivery catheters employed, particularly with balloon-expandable technologies, procedures were undertaken with general anaesthesia with surgical cut down for femoral access. Intraprocedural transoesophageal echocardiographic guidance was the norm[6, 7]. However, TOE carries a risk of oesophageal injury and increases procedure times[8, 9]. Since 2007 there has been serial reduction in the entry profile of delivery devices for THVs such that a greater proportion of patients are suitable for transfemoral procedures. Over 90% of cases in the UK are now undertaken with percutaneous, transfemoral access under conscious sedation. This shift in practice from general anaesthesia to conscious sedation has been accompanied by a transition from TOE to TTE as the intraprocedural imaging modality of choice.

## Role of intraprocedural TTE

Intraprocedural echocardiographic imaging is used principally to assess prosthetic valve function post-deployment and to allow early detection of complications. There are 3 distinct phases where TTE is employed in TAVI procedures:

1. On-table pre-procedure assessment
2. Immediate post THV deployment assessment
3. Pre-cath lab exit assessment

### On-table pre-procedure assessment

**Fig. 1:**
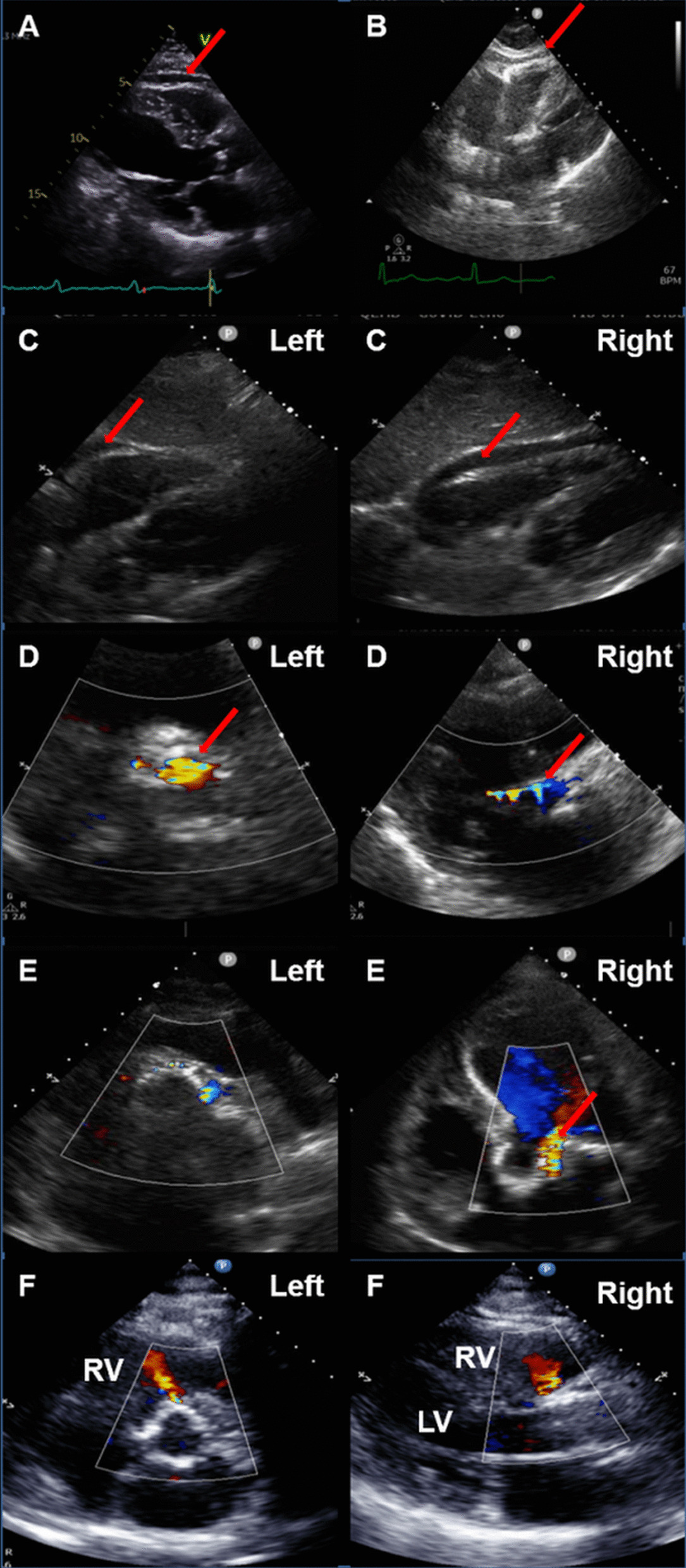
**A** Baseline echocardiogram showing small < 1 cm pericardial effusion (arrow). **B** A pericardial fat pad was noted on the baseline echocardiogram (arrow). This may be confused with a pericardial effusion on the post-deployment study. **C** A small pericardial effusion (arrow) was noted on the baseline study (Left). Post THV deployment the effusion enlarged (arrow) and required emergency pericardiocentesis (Right). **D** Long axis view showing wire-induced AR (arrow). The colour Doppler jet often ‘hugs’ the wire (Right). On the short axis view the wire-induced AR lies within the frame (Left) and is adjacent to the stiff wire (arrow). **E** On short axis a moderate jet of PVR can be seen at 3 o’clock with trivial jets at 10–12 o’clock. The jets lie outside the THV frame (Left). Apical 5 chamber view showing the PVR jet (arrow) outside the frame (Right). **F** A interventricular VSD was noted after deployment of a balloon expandable THV. The VSD was visible on the short axis (left) and long axis (right)

A rapid assessment should be made whilst preparations are underway for gaining vascular access. The value of this is to allow a baseline study for comparison with changes that may develop during the procedure. The location of optimal echocardiographic windows should be carefully noted in order to improve quality and avoid suboptimal images which may delay the procedure and hamper accurate assessment. Where necessary, slight tilting of the patient with a wedge under the right chest may be employed to improve echocardiographic image quality. Careful positioning at the start of the procedure is important as the patient may be unable to move and reposition themselves during the procedure. Additionally, image acquisition should be done with ECG-triggering as standard (Fig. 1A). This ensures measurements are taken during the correct phase of the cardiac cycle. This is particularly important when documenting the size of pericardial effusion, which should be assessed at end-diastole (Fig. 1A) or LVOT diameter measurement, which should be taken at mid-systole. To ensure standardization of measurements, it is recommended that the Nyquist limit should be set between 50 and 60 cm/s and where appropriate by reducing colour flow Doppler region of interest in order that colour flow frame rates are kept above 20/s. An evaluation of the pericardial space is particularly important. Presence of pre-existing pericardial effusion (Fig. 1A) and epicardial fat (Fig. 1B) that may subsequently be confused for a new pericardial effusion should be noted. Views should be obtained from the subcostal, left-parasternal and apical windows. An assessment should also be made of left ventricular function and the presence and severity of mitral regurgitation should also be noted. An in-depth assessment of these parameters is not required as the principal purpose of the examination is to allow rapid identification of changes. The entire assessment should take less than 5 min to complete.

### Immediate post THV deployment assessment

Immediately post THV deployment echocardiography should be undertaken to rapidly identify complications, to assess prosthesis function and paravalvular regurgitation.

#### Pericardial effusion

The first imaging window is the subcostal region to assess for development of pericardial effusion which may indicate injury to the annular complex or LVOT (1C Right). A comparison with the immediate pre-procedure images is important to help determine new changes (1C Left, Additional file 1: Video S1). Pericardial effusion noted on echo is often visible before haemodynamic changes develop. If new pericardial effusion or a change in size of a pre-existing effusion is noted, careful attention should be given to haemodynamic indicators of tamponade and the effusion should be carefully monitored over several minutes. If a growing pericardial effusion is noted equipment should be readied for emergency pericardiocentesis and aortography undertaken to detect a bleeding source. The surgical team may need to be alerted to consider the need for emergency sternotomy.

#### Aortic regurgitation

Both paravalvular (PVR) and transvalvular aortic regurgitation (TAR) may be seen post THV deployment. Assessment should be made from short axis, left parasternal long axis and apical windows. TAR may be artefactually caused by the stiff catheter delivery wire. A transverse, short-axis view just below the THV in the LVOT is particularly useful in differentiating wire-induced TAR from PVR (Additional file 2: Video S2). Wire-induced TAR will be seen ‘hugging’ and adjacent to the wire (Fig. 1D Right). The left parasternal long axis view and apical three and five chamber views are also useful and turbulent flow on colour Doppler may be identified around the wire (Fig. 1D Left, Additional file 3: Video S3).

PVR arises from outside the THV frame and is best identified on the short axis LVOT view, just below the THV (Fig. 1E Left, Additional file 4: Video S4) and may also be seen on the parasternal long axis, apical three and five chamber views (Fig. 1E Right, Additional file 5: Video S5). Moderate or severe, but not mild or trivial, PVR is associated with increased 1 year mortality after TAVI [10, 11]. Identification of moderate or severe PVR may guide further balloon valvuloplasty to reduce paravalvular regurgitation with both balloon-expandable and self-expanding valves.

#### Ventricular function

Coronary obstruction may complicate THV deployment and is seen in approximately 1% of cases. The left main coronary artery is most often involved and females are affected more commonly [12]. The resulting ischaemia may cause global or regional wall motion abnormalities, which are usually associated with evidence of ischaemia on the ECG and hypotension. If new global or regional left ventricular dysfunction is seen a root aortogram or selective coronary angiogram should be immediately undertaken.

#### Ventricular septal defects

A rare complication of TAVI is iatrogenic ventricular septal defect with an incidence of < 0.4%[13]. Interventricular defects are most common (Fig. 1F, Additional file 6: Video S6, Additional file 7: Video S7) with LV-RA defects being rarer. They are thought to arise as a result of focal annular rupture[14]. They may be asymptomatic or cause heart failure. Small and asymptomatic defects may be managed conservatively but larger defects may warrant surgical or percutaneous closure.

#### Damage to mitral valve apparatus

Damage to sub-valvular mitral apparatus may result in mitral regurgitation and a comparison with the pre-procedure images is important to determine whether mitral regurgitation is new or pre-existing. Sub-valvular apparatus damage can occur when the guide wire inadvertently passes through the mitral valve apparatus leading to subsequent damage of the chordae by the valve or injudicious wire removal.

Entanglement of the stiff guide wire can be assessed with TOE, however, the fluoroscopic appearances and movement of the guide wire have largely superseded echocardiographic identification of entanglement with the sub valvular mitral apparatus.

### Post procedure pre lab-exit echocardiogram

A focused echocardiogram should be undertaken before leaving the catheterization laboratory. Repeat assessment is undertaken to ensure that no pericardial effusion has developed since the immediate post THV deployment study. Assessment is made of the aortic valve and parameters are gained for measurement of aortic valve area, peak and mean gradient and a final assessment of aortic and mitral regurgitation and LV function. This obviates the need for a further routine TTE on the ward in the absence of a clinical change, thereby reducing costs and the burden on echocardiography services.

## Conclusion

**Fig. 2:**
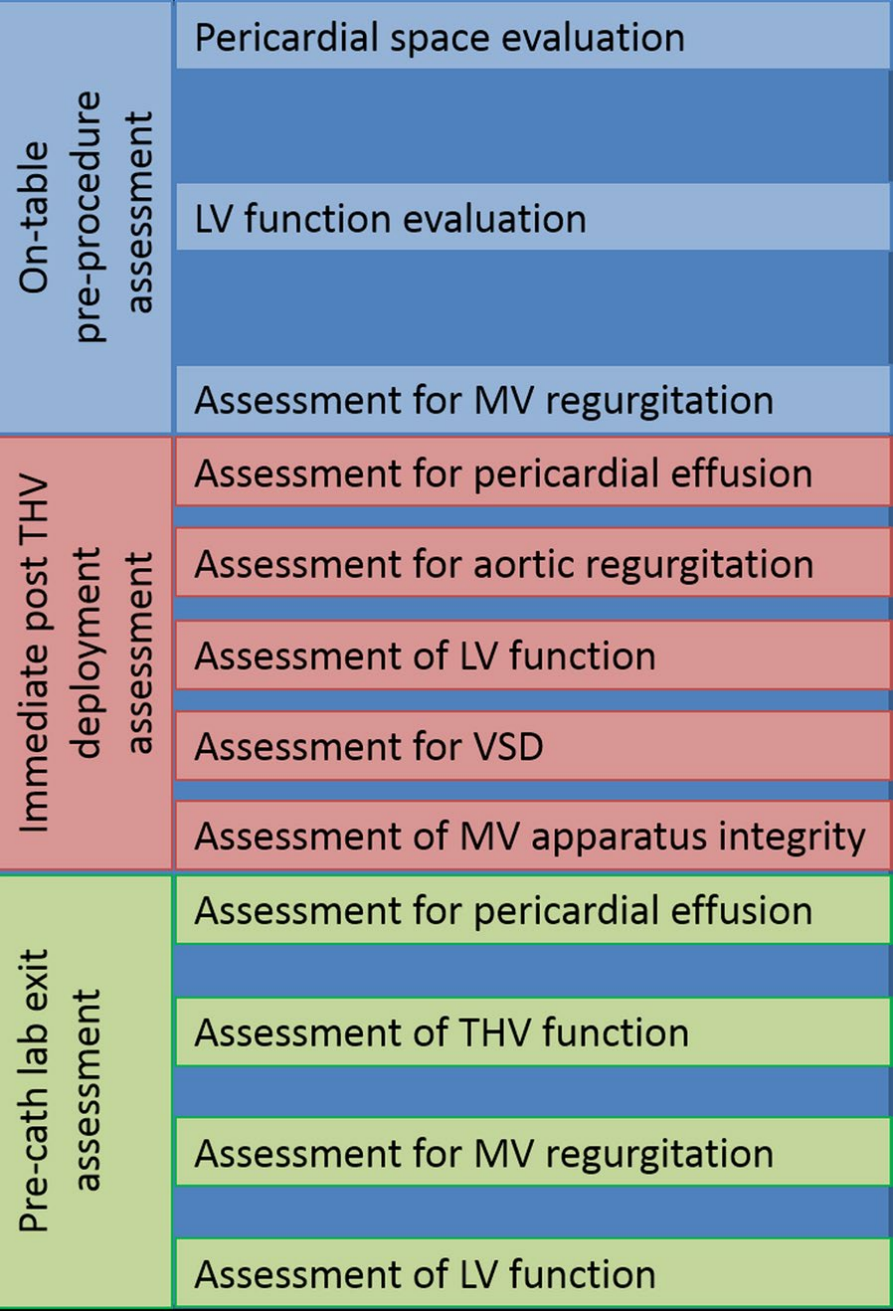
Assessment flow chart

With the continued growth of TAVI undertaken with conscious sedation rather than general anaesthesia, TTE has become the dominant periprocedural echocardiographic modality[15]. Echocardiographers should exercise a time-efficient protocol involving on-table baseline assessment, immediate post-deployment assessment and a final pre-lab exit study (Fig. 2). Early recognition of periprocedural complications using echocardiography permits timely intervention and may improve outcomes.

## Supplementary Information

**Additional file 1: Video S1:** Subcostal view post THV deployment showing a new pericardial effusion with RV invagination.

**Additional file 2: Video S2:** Short axis view showing trans valvular aortic regurgitation (12 o’clock). Jet lies within the THV frame and adjacent to the delivery wire.

**Additional file 3: Video S3:** Apical 5 chamber view showing TAR jet within the THV frame and ‘hugging’ the delivery wire.

**Additional file 4: Video S4:** Small PVR jets at 12 o’clock. Larger PVR jet at 3 o’clock. PVR lies outside the THV frame.

**Additional file 5: Video S5:** Apical 5 chamber view showing PVR jet outside the THV.

**Additional file 6: Video S6:** Parasternal long axis view showing small VSD post deployment of balloon expandable THV.

**Additional file 7: Video S7:** Short axis view at LVOT level showing VSD.

## Data Availability

Not applicable.

## Abbreviations

TAVI: Transcatheter aortic valve implantation
TOE: Transoesophageal echocardiography
THV: Transcatheter heart valve
TTE: Transthoracic echocardiography
